# Human Amnion-Derived Mesenchymal Stromal Cells: A New Potential Treatment for Carbapenem-Resistant *Enterobacterales* in Decompensated Cirrhosis

**DOI:** 10.3390/ijms23020857

**Published:** 2022-01-13

**Authors:** Mariangela Pampalone, Giampiero Vitale, Salvatore Gruttadauria, Giandomenico Amico, Gioacchin Iannolo, Bruno Douradinha, Alessandra Mularoni, Pier Giulio Conaldi, Giada Pietrosi

**Affiliations:** 1 Ri.MED Foundation, 90133 Palermo, Italy; givitale@fondazionerimed.com (G.V.); gamico@fondazionerimed.com (G.A.); bdouradinha@nykode.com (B.D.); 2 Department of Laboratory Medicine and Advanced Biotechnologies, IRCCS-ISMETT (Mediterranean Institute for Transplantation and Advanced Specialized Therapies), 90127 Palermo, Italy; giannolo@ismett.edu (G.I.); pgconaldi@ismett.edu (P.G.C.); 3 Department for the Treatment and Study of Abdominal Disease and Abdominal Transplantation, IRCCS-ISMETT, UPMC, 90127 Palermo, Italy; sgruttadauria@ismett.edu (S.G.); gpietrosi@ismett.edu (G.P.); 4 Department of General Surgery and Medical-Surgical Specialties, University of Catania, 95124 Catania, Italy; 5 Unit of Infectious Diseases, IRCCS-ISMETT, 90127 Palermo, Italy; amularoni@ismett.edu

**Keywords:** cirrhosis, ascitic fluid, spontaneous bacterial peritonitis, human amnion-derived mesenchymal stromal cells, carbapenem-resistant Enterobacterales, pattern recognition molecules, ficolins, complement, placenta

## Abstract

Background: Spontaneous bacterial peritonitis (SBP) is a severe and often fatal infection in patients with decompensated cirrhosis and ascites. The only cure for SBP is antibiotic therapy, but the emerging problem of bacterial resistance requires novel therapeutic strategies. Human amniotic mesenchymal stromal cells (hA-MSCs) possess immunomodulatory and anti-inflammatory properties that can be harnessed as a therapy in such a context. Methods: An in vitro applications of hA-MSCs in ascitic fluid (AF) of cirrhotic patients, subsequently infected with carbapenem-resistant Enterobacterales, was performed. We evaluated the effects of hA-MSCs on bacterial load, innate immunity factors, and macrophage phenotypic expression. Results: hA-MSCs added to AF significantly reduce the proliferation of both bacterial strains at 24 h and diversely affect M1 and M2 polarization, C3a complement protein, and ficolin 3 concentrations during the course of infection, in a bacterial strain-dependent fashion. Conclusion: This study shows the potential usefulness of hA-MSC in treating ascites infected with carbapenem-resistant bacteria and lays the foundation to further investigate antibacterial and anti-inflammatory roles of hA-MSC in in vivo models.

## 1. Introduction

Spontaneous bacterial peritonitis (SBP) is the most relevant infectious complication, developing in 10–25% of patients with decompensated cirrhosis with ascites [1,2], causing about 5–25% mortality in cirrhotic patients awaiting liver transplantation [3]. The most important pathogenic mechanism for SBP is bacterial translocation and the most common responsible organisms are Enterobacterales, Escherichia Coli (E. coli) being the major pathogen, followed by Klebsiella pneumoniae (K. pneumoniae) and Enterococcus faecium [4]. Nowadays, SBP, due to multidrug-resistant microorganisms (MDR) and extensively drug-resistant organisms (XDR), is a complex healthcare problem and is responsible for a poor prognosis and high mortality rate in cirrhotic patients [5]. Regardless of the mechanism through which bacteria reach the ascitic fluid (AF), the probability of developing an infection is inversely proportional to the bactericidal capacity of the ascites themselves, through the activation of humoral bactericidal mechanisms, mainly the complement system [6]. This system includes three activation pathways; the classical pathway, the lectin pathway, and the alternative pathway [7]. The development of SBP is essentially based on protein concentration in AF, on complement factors, and on opsonic activity [8]. In decompensated cirrhosis, several mechanisms contribute to immune dysfunction and, in particular, the specific immunity in AF is deeply modified for a reduced attractive activity and a reduced expression of complement system factors (C4 and C3), which makes the first immune defense mechanism ineffective [9,10]. Furthermore, mannose, binding lectin (MBL) and ficolins (ficolin 1, ficolin 2, and ficolin 3), is a soluble pattern recognition molecule (PRM), which, in the lectin complement pathway, acts as a mediator of host defense [11] and is incorrectly produced in patients with cirrhosis, increasing the risk of bacterial infections [12]. We have previously shown that human amnion-derived mesenchymal cells (hA-MSCs) are able to increase anti-inflammatory cytokines and induce an M2-biased polarization in AF macrophages. Additionally, M1 macrophage levels did not decrease significantly in the presence of hA-MSCs, confirming the ability of hA-MSCs to find a balance in the resolution of the pathological condition [13]. Moreover, a significant increase in TNF-α release was shown, starting from 72 h, and was retained up to 1 week [13].

The aim of this study was to evaluate the therapeutic effect of hA-MSCs in the treatment of Enterobacterales-infected ascites, which were obtained from cirrhotic patients and exposed in vitro to carbapenem-resistant Enterobacterales (CRE).

In particular, we evaluated the therapeutic role of hA-MSCs in bacterial clearance and macrophage phagocytosis, as well as their crosstalk with the immune cells present in AF and their influence on the PRMs of the lectin-complement pathway and complement protein production.

## 2. Results

### 2.1. Determination of Bacterial Load after hA-MSCs Co-Culture Shows a Better Trend in AF Infected with Carbapenem-Resistant Enterobacterales

**Figure 1:**
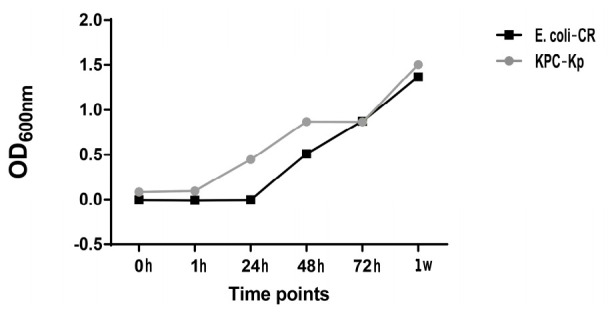
Post-paracentesis AF infection with Carbapenem-resistant Enterobacterales time course, without hA-MSCs. The proliferation of KPC-Kp bacteria begins its exponential phase after 1 h of treatment; for E. coli-CR, proliferation starts after 24 h.

A preliminary experiment was carried out in order to evaluate the in vitro contamination time course of both CRE bacteria, E. coli NDM-1/OXA-48 (E. coli-CR) and a K. pneumoniae KPC-3 clinical isolate (KPC-Kp) (Figure 1). The representative graph clearly shows how in vitro infection by KPC-Kp begins to increase bacterial proliferation starting from 1 h, while for E. coli-CR it increases from 24 h.

**Figure 2:**
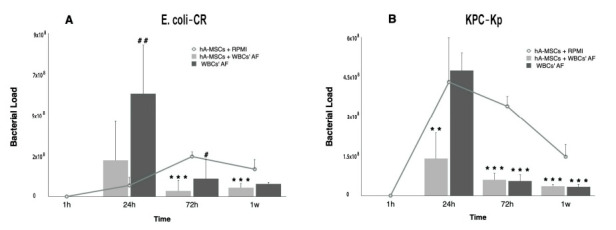
The effects of hA-MSCs on the bacterial load of AF infected with E. coli-CR (**A**) and KPC-Kp (**B**). The bacterial proliferation after 1 h, 24 h, 72 h, and 1 week of exposure, compared with standard culture in RPMI (*), and comparing AF cultured with or without hA-MSCs (#) during the same time-point set. (**A**) A statistically significant increase of bacterial load was seen after 24 h of exposure to WBCsAF (*p* < 0.01, ##) while no significance was shown both conditions compared to RPMI. However, starting at 72 h, a decrease in bacterial load in hA-MSCs-WBCs was significantly evident compared with RPMI (72 h and 1 week, both with *p* < 0.001, ***), while there was no significant decrease in bacterial proliferation in AF without hA-MSCs, when compared to RPMI. At 72 h, a significant increase in bacterial proliferation was seen in AF samples not treated with hA-MSCs (*p* < 0.05, #). (**B**) Decrease in bacterial proliferation, under the same culture conditions at different time points, was shown at 72 h and at 1 week in both types of samples, compared with RPMI (*p* < 0.001, ***). The bacterial load grown in hA-MSCs plus WBCsAF was reduced after 24 h (*p* < 0.01, **), while there was an increase in the bacterial load in the AF not treated with hA-MSCs. Values are expressed as numeric means ± standard deviation (SD).

To determine the ability of hA-MSCs to impair Enterobacterales growth levels, AF collected from cirrhotic patients was infected with the aforementioned microorganisms, and the bacterial loads were quantified at different time points. As controls, we used hA-MSC grown in RPMI without antibiotics (Control 1) and white blood cell AF (WBCsAF) without hA-MSCs (Control 2); the latter was to evaluate how the presence of WBCs in AF may affect its capability to inhibit bacterial growth. The co-culture of hA-MSCs with WBCsAF showed, at some time points, a significant inhibition of bacterial growth with respect to both controls. In particular, we observed some differences in terms of response against the two CRE strains (Figure 2). E. coli-CR proliferation was significantly reduced at 72 h (*p* < 0.001) and at 1 week (*p* < 0.001) post infection in the AF samples containing hA-MSCs compared to the bacterial growth measured in both controls (Figure 2A). Furthermore, we observed a progressive bacterial load increase in Control 2 at 24 (*p* < 0.01) and 72 h (*p* < 0.05), when compared to co-culture conditions with hA-MSCs (Figure 2A). KPC-Kp proliferation was reduced at 24 h (*p* < 0.01), at 72 h (*p* < 0.001), and at 1 week (*p* < 0.001) in hA-MSCs plus WBCsAF samples, when compared to the control (Figure 2B). Moreover, in Control 2, with respect to Control 1, a significant reduction in bacterial load was assessed at 72 h (*p* < 0.001) and 1 week (*p* < 0.001).

### 2.2. Effects of hA-MSCs on Macrophage Phenotypes in Presence of Carbapenem-Resistant Enterobacterales

Macrophages are important components of innate immunity and play a major role in cell homeostasis maintenance and the host cellular defense system by modulating the inflammatory response and phagocytosis [14,15]. Macrophages can adopt different functional phenotypes according to the surrounding environment, including a classically activated phenotype (M1) and an alternative activated phenotype (M2) [13,16]. M1 macrophages are characterized by a production of pro-inflammatory cytokines, chemokines, and reactive oxygen species (ROS) [17]. Conversely, M2 macrophages are characterized by a production of anti-inflammatory cytokines, chemokines, and activation of antioxidant and anti-inflammatory signaling pathways, thus favoring tissue healing and a return to homeostasis [17,18,19,20]. In our previous work [13], we used LPS to mimic an uncomplicated ascites infection scenario, and demonstrated the ability of hA-MSCs to increase M2-like skewing at 72 h (compared to 1 and 24 h), which decreased after 1 week. Moreover, we noted that the M1-like component, in the presence of hA-MSCs, was significantly higher at 1 week with respect to 24 h, demonstrating that hA-MSCs may influence both macrophage populations present in the AF of cirrhotic patients. This suggests that macrophages, in presence of hA-MSCs, may reacquire their phagocytic properties and, consequently, eliminate bacteria that translocate into the AF in advanced cirrhosis, and decrease posteriorly (by reverting to M2-like macrophages phenotype) with resolution of the infection.

The main objective of this work was to assess the therapeutic effects of hA-MSCs added to AF infected by carbapenem-resistant Enterobacterales, and the antimicrobial phagocytic activity of macrophages present in the same, up to 1 week post incubation.

**Figure 3:**
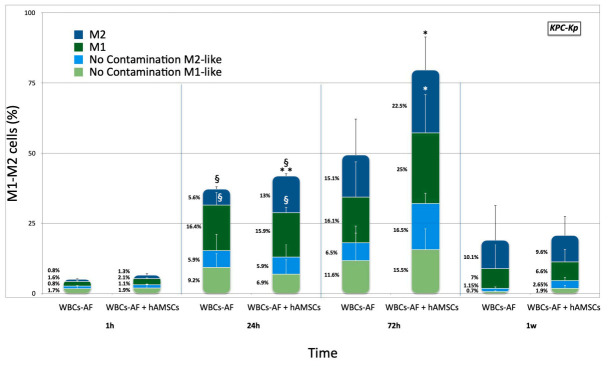
M1 and M2 phenotypes expressed by WBCs derived from the ascites of cirrhotic patients infected with KPC-Kp after co-culture, with and without hA-MSCs, for the time points chosen (1 h, 24 h, 72 h, and 1 week) compared to the M1 and M2 phenotypes expressed by WBCs cultured without KPC-Kp. The graph shows that there were no significant differences in the expression of the M1 and M2 components in samples not infected with KPC-Kp, regardless of the presence of hA-MSCs in culture. However, in the presence of bacteria, at 24 h of co-culture, a significant increase of both M1- and M2-like cells was observed compared to 1 h (*p* < 0.05, §), both in the presence of hA-MSCs and in Control 2, while a boosted reaction of the M2 component occurred in the presence of hA-MSCs compared to Control 2 at the same time-point (*p* < 0.01, **). At 72 h, an increase in the M1 and M2 components in the presence of hA-MSCs can be observed, compared to Control 2 (*p* < 0.05, *).

**KPC-Kp.** To determine the effect of KPC-Kp on the induction of the M1 functional phenotype, a series of experiments were performed to directly compare surface expression of maturation markers. Unstimulated AF macrophages isolated from patients after paracentesis were cultured with bacteria, at all time points, and were tested with and without hA-MSCs in order to evaluate if the presence of hA-MSCs is involved in the development of the M1 inflammatory phenotype in a clinically relevant infection. The WBCs were analyzed using flow cytometry to determine the state of macrophage polarization, before and after direct co-culture with hA-MSCs and KPC-Kp, at 1, 24, 72 h, and at 1 week. The prevalence of CD14+ CD16+ M1-like with respect to CD14+ CD206+ M2-like macrophages constantly changed during co-culture, using the 1 h time point as reference (Figure 3). In fact, from 1 to 24 h, a significant increase of M1 (*p* < 0.05) and M2 (*p* < 0.05) populations was seen in presence of hA-MSCs and in Control 2, but at 24 h we observed a drastic and significant increase in the M2 population (*p* < 0.01) compared to Control 2. Moreover, after 72 h of co-culture with hA-MSCs, an M2 component rise was confirmed and a drastic increase in M1-like macrophages was also seen in comparison to Control 2 (*p* < 0.05), confirming the results of our previous work and supporting the hypothesis that mesenchymal stromal cells obtained from human placenta have rapid (within 3 days) anti-inflammatory and immunomodulatory actions.

**Figure 4:**
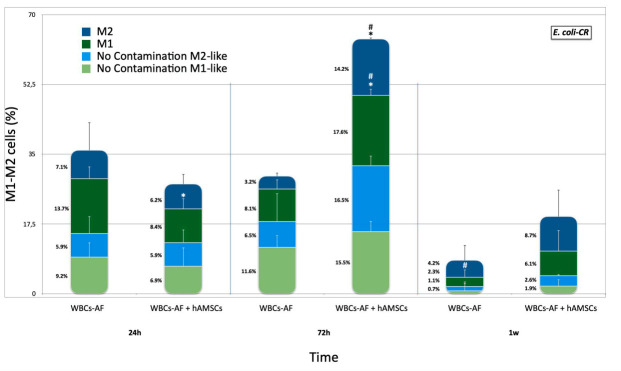
M1 and M2 phenotypes expressed by WBCs derived from the ascites of cirrhotic patients infected with E. coli-CR, after co-culture, with and without hA-MSCs, for the time points chosen (1 h, 24 h, 72 h, and 1 week) compared to the M1 and M2 phenotypes expressed by WBCs cultured without E. coli-CR infection. The macrophage polarization was influenced after 72 h in the presence of hA-MSCs. In particular, at 24 h, there was a reduction in M1 macrophage (*p*  <  0.05, *) in the presence of hA-MSCs compared to M1 macrophage grown without hA-MSCs under the same conditions but, at 72 h, the presence of MSCs showed a significant increase in both M1-like and M2-like components compared to the same culture conditions, both at 24 h (*p*  <  0.05, #) and at the same time in the absence of MSC (*p*  <  0.05, *). At 1 week, a significant reduction in M1 macrophage population was found compared to 24 h (*p* < 0.05, #), while there was no change in both M1 and M2 macrophage populations grown in co-culture with hA-MSC.

**E. coli-CR.** Unstimulated AF macrophages, isolated from patients after paracentesis, were cultured with E. coli-CR and tested, with and without hA-MSCs, at 24, 72 h, and at 1 week. The WBCs were analyzed by flow cytometry to determine the state of macrophage polarization before and after direct co-culture with hA-MSCs and E. coli-CR at all time points (Figure 4), as described above. At 72 h of co-culture, an increase in M1-like macrophages in the presence of hA-MSCs was seen compared with Control 2 (*p* < 0.05). A significant increase in both M1 and M2 phenotypes was shown at 72 h, in co-culture with mesenchymal cells, compared to the same component at 24 h (*p* < 0.05).

### 2.3. Phagocytic Capacity of the WBCs’ AF Component in Co-Culture with hA-MSCs

**Figure 5:**
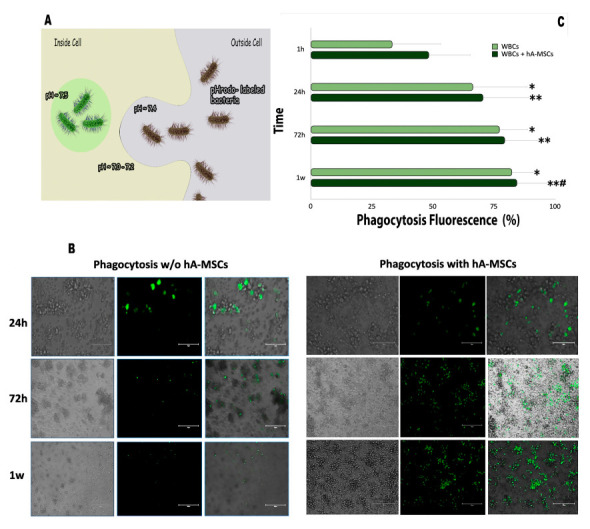
(**A**) A representative image for phagocytosis of pHrodo-labeled E. coli bioparticles, which are nonfluorescent at neutral pH, but fluorescent (bright green) in acidic environments. This increase in fluorescence signal is directly correlated to engulfment of pH sensitive dye-conjugated bioparticles and occurs at low pH. (**B**) Detection of phagocytosis cells using pHrodo™ Green E. coli BioParticles^®^. The panel of fluorescence pictures shows WBCsAF grown, with or without hA-MSCs, after 1 h, 24 h, 72 h, and 1 week. Fluorescence can be observed from 72 h onwards in presence of hA-MSCs, indicating high levels of phagocytosis, when compared to samples not treated with hA-MSCs. Scale bar, 150 μm. (**C**) Effects of hA-MSCs on phagocytosis of E. coli BioParticles by WBCsAF. Statistics were obtained comparing with 1 h (*) and 24 h (#). Values were statistically significant when *p*  <  0.05 (*, #), *p*  <  0.01 (**), and are expressed as means of percentages and SD. Phagocytosis of the particles by WBCs was measured by flow cytometry and the fluorescence signal generated was proportional to the amount of phagocytosis. We can see that WBCs cultured without hA-MSCs engulfed the bioparticles with a significant fluorescence increase (*p*  <  0.05,*) at all time points, when compared to 1 h, while, in the presence of hA-MSCs, an increase in fluorescence is observed at all times, when compared to 1 h (*p*  <  0.01, **) and 24 h (*p*  <  0.05, #).

Phagocytes play a critical role in host defense against extracellular bacteria [19]. We have previously shown a WBCsAF marked flexibility in phagocytic activity when co-cultured with hA-MSCs, both in terms of expression of the characteristic markers of the M1 phenotype and in a reduction of post-culture bacterial load. We investigated the effects of hA-MSCs on the phagocytic activity of monocytes through flow cytometry analyses and fluorescence microscopy using pHrodo green-conjugated E. coli bioparticles. These bioparticles are non-fluorescent outside the leukocytes at neutral pH, but bright green fluorescent at acidic pHs, such as in phagosomes (Figure 5A). In combination with fluorescence microscopy (Figure 5B), phagocytosis by mature monocytes was quantified (Figure 5C). As shown in Figure 5B, the amount of E. coli bioparticles, which were engulfed (green) by control monocytes, was smaller in comparison to the number of bacterial particles present within monocytes cultured with hA-MSCs. Moreover, there was a clear difference in the fluorescence intensity of E. coli bioparticles phagocyted by monocytes cultured with hA-MSCs. Figure 5C shows the intensity of fluorescence in a time-dependent fashion, confirming that the presence of mesenchymal stromal cells reduced the quantity of bacteria, in accordance with the fluorescence images (Figure 5B). At 24, 72 h, and 1 week, the fluorescence intensity detected in WBCs plus hA-MSCs was greater (*p* < 0.01) than that shown by the WBCs-only group (*p* < 0.05). In agreement with these results, there was a significant increase in the percentage of fluorescence in the WBCs + hA-MSCs group, between 24 h and 1 week.

### 2.4. Mesenchymal Stromal Cells Induce Greater Activation of the Complement System with a Lower Formation of Pro-Inflammatory Molecules

**Figure 6:**
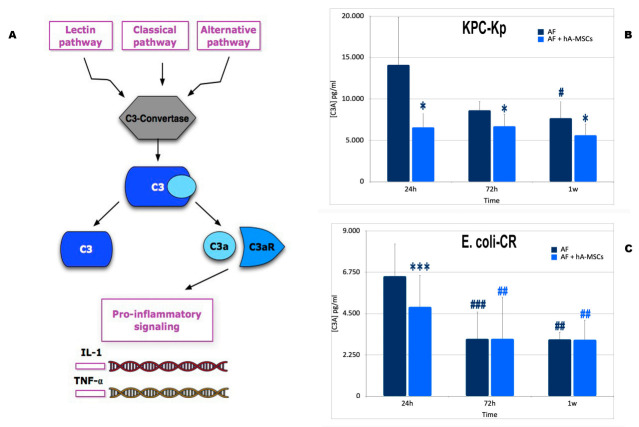
(**A**) The figure shows a very simplified view of the complement system. The complement system can be initiated by three different pathways: the classic pathway, the lectin pathway, and the alternative pathway, all converging on the formation of C3 convertases. This classic C3 convertase activates and cleaves C3 molecules into C3b and C3a. C3a is a critical chemotactic mediator that exerts various effects after binding to the C3a receptor (C3aR). C3a/C3aR plays pro-inflammatory effects, upregulating the expression of proinflammatory mediators releasing extracellular ATP in monocytes or macrophages and promoting T cell proliferation. (**B**) C3A production during AF’ infection with KPC-Kp cultured with or without hA-MSCs for 24 h, 72 h, and 1 week. Significant decrease was shown only during culture with hA-MSCs after 24 h, 72 h and 1 week (*p* < 0.05, *) compared to Control 2. WBCs cultured without hA-MSCs (Control 2) showed a significantly decrease at 1 week compared to 24 h (*p* < 0.05, #). (**C**) C3A production during AF infection with E. coli-CR cultured with and without hA-MSCs for 24 h, 72 h, and 1 week. A significant decrease of AF plus hA-MSCs was shown only at 24 h (*p* < 0.001, ***) compared to Control 2 at the same time. While, both at 72 h and at 1 week, there were no significant changes in C3a production in the presence or absence of hA-MSCs at the same analyzed time points. The data obtained showed a significant decrease in C3A at 72 h and at 1 week in both samples analyzed compared to 24 h (Control 2 at 72 h, *p* < 0.001, ###; at 1 week, *p* < 0.01, ##; WBCsAF plus hA-MSCs, at both 72 h and 1 week (*p* < 0.01, ##).

There are three major ways of complement activation: the alternative pathway, the lectin pathway, and the classical pathway. In all three pathways, the central event of complement activation is the cleavage of the C3 protein [21,22,23,24]. The three activation pathways converge in a common final target, when the C3 convertase divides C3 into C3a and C3b [25,26,27], C3a, also called anaphylatoxin, is a small peptide fragment that exerts pro-inflammatory and chemotactic activities and mediates the activation and recruitment of leukocytes [28] and pro-inflammatory cytokine production, as shown in Figure 6A.

In the presence of pathogenic insult, a significant decrease in C3a, compared to the ascitic medium cultured without hA-MSCs, both at 24 and 72 h, and at one week, was observed. In the presence of KPC-Kp, there was a significant reduction of C3a production, at all times, compared to the quantity produced in AF deprived of hA-MSCs (*p* < 0.05) (Figure 6B). On the other hand, in the presence of carbapenem-resistant Enterobacterales infection, in particular E. coli-CR infection, a significant reduction in C3a was detected in the ascitic medium in co-culture with hA-MSCs compared to Control 2 (AF not treated with hA-MSCs), at 24 h (*p* < 0.001) (Figure 6C). However, at 72 h and at 1 week, there was a significant decrease compared to 24 h in both groups. In particular, the decrease in anaphylatoxin production was significant in both groups, at 72 h and at 1 week, compared to 24 h (at 72 h, Control 2 was *p* < 0.001 and AF plus hA-MSCs was *p* < 0.01; at 1 week, both Control 2 and AF plus hA-MSCs were *p* < 0.01). Thus, our results show how the presence of mesenchymal stromal cells, obtained from human placenta, can reduce the production of the pro-inflammatory component C3a.

### 2.5. Levels of Pattern Recognition Molecules (PRMs), Mannan-Binding Lectin (MBL) and Ficolins (FNCs)

**Figure 7:**
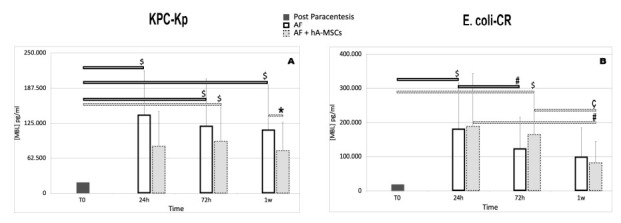
Mannose-binding lectin analysis using WBCs derived from ascites of cirrhotic patients co-cultured, with or without hA-MSCs, for the time point chosen (24 h, 72 h, and 1 week) after KPC-Kp (**A**) and E. coli-CR infection (**B**). Statistics were obtained comparing with T0 ($), 24 h (#), and 72 h (ç) and comparing AF plus hA-MSCs versus Control 2 at the same time (*). (**A**) WBCsAF grown without hA-MSCs (Control 2) had a significant increase in MBL at all times, compared to T0 (*p* < 0.05, $). Interestingly, not only did the MBL concentration in presence of hA-MSCs increase significantly only at 72 h with respect to T0, but its concentration was also significantly lower at 1 week compared to WBCs without hA-MSCs. (**B**) The hA-MSC effect was significantly relevant at 1 week, as there was a decrease in MBL compared to both 24 h and 72 h (*p* < 0.05, ç #). Only at 72 h the WBCs plus hA-MSCs condition showed a significant increase in MBL production compared to T0, while Control 2 shows a significant increase at 24 h compared to T0 (*p* < 0.05, $) and a significant decrease at 72 h when compared to 24 h (*p* < 0.05, #), which remained unchanged after 1 week. Values were statistically significant when *p* < 0.05 (*, $, #, ç) and are expressed as means of MBL concentration in pg/mL (*n* = 6) and SD.

The lectin pathway of the complement system is initiated when mannan-binding lectin (MBL) or ficolins, in complex with the three MBL-associated serine proteases (MASPs: MASP-1, MASP-2 and MASP-3) and MBL-associated proteins (MAp19 and MAp44), recognize microbial polysaccharides [29]. Previous studies have reported increased expression of MBL in response to inflammatory stimuli or in the presence of infections [30,31,32]. In this study we aimed to investigate the presence of soluble PRMs of the lectin–complement pathway and complement proteins in the AF of patients with cirrhosis. In particular, the main goals were to quantify the concentration of MBL, Ficolin 2, and Ficolin 3 in AF and subsequently, after in vitro infection with carbapenem-resistant Enterobacterales, as E. coli-CR and KPC-Kp strains, to monitor the trend of the concentrations of the same proteins over time in the presence (or not) of hA-MSCs. As shown in Figure 7A, following the stimulus of infection with KPC-Kp, the concentration of MBL increased significantly in AF not treated with hA-MSCs compared to the initial time, i.e., the concentration of MBL post paracentesis (*p* < 0.05). While the trend of MBL concentration in AF treated with the mesenchymal stromal cells showed a significant increase only at 72 h (*p* < 0.05), compared to the zero time, a significant decrease at 1 week compared to AF at the same time (*p* < 0.05) was observed. Infection with E. coli-CR instead is represented in Figure 7B. In this case, it is interesting to note that, at 1 week, hA-MSCs are able to induce a significant decrease in the concentration of MBL compared to the same group, both at 24 h and at 72 h (*p* < 0.05), while there is no significance in AF without hA-MSCs. At 24 and 72 h, the AF component shows a significant increase with respect to 0 h (*p* < 0.05) and 24 h, respectively. The AF plus hAMSC component at 72 h shows a significant increase (*p* < 0.05) compared to 0 h. The data obtained suggest that the reduction in the concentration of MBL at one week in the AF plus hA-MSCs group, for both bacterial species, could be due to a higher usage of the available MBL protein present in the medium, as confirmed previously by the decrease in bacterial load. These results are also in agreement with a previous work, which shows that circulating PRMs in the lectin pathway are associated with several disease activities [33].

The FNCs are a group of oligomeric lectin proteins that recognize components of bacterial or fungal cell walls. The role of ficolins in inflammatory diseases has not yet been fully clarified because there are several conflicting data in the literature that do not clarify how the trend of ficolins can be correlated to different pathologies [33,34]. In this study, we investigated whether, in this regard, the use of hA-MSCs cells could help us to better understand the role of ficolins. However, we found no significant changes that indicate that the presence of hA-MSC has further, higher roles in complement activation. The concentration of ficolin 2 shows a significant increase at 72 h and at 1 week with respect to 0 h, both in the case of infection with E. coli-CR and with KPC-Kp (*p* < 0.05). There are no significant differences between the two different groups at the same time.

**Figure 8:**
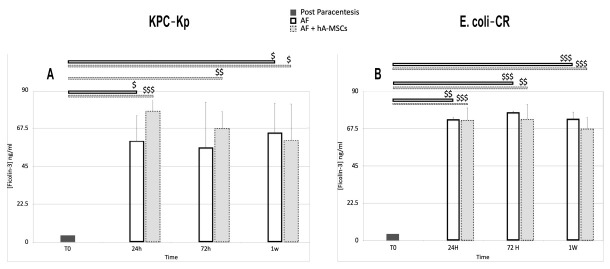
Ficolin-3 levels assessed using ELISA during AF infection with KPC-Kp (**A**) and E. coli-CR (**B**) cultured, with and without hA-MSCs, for 24 h, 72 h, and 1 week. Statistical analysis was done by comparison of results with T0 initial data ($). The data obtained showed a higher quantity of ficolin 3 at all time points, with respect to T0, for both types of infection. In particular, the presence of KPC-Kp determined a more relevant significance at 24 and 72 h in the presence of hA-MSCs (respectively, *p* < 0.001, $$$ and *p* < 0.01, $$) which decreased up to 1 week (*p* < 0.05, $). The absence of hA-MSCs resulted in a lower significance only at 24 h and 1 week (*p* < 0.05, $). (**B**) The E. coli-CR infection does not show a substantial difference in terms of ficolin 3 quantified in the two types of samples treated.

The trend of ficolin 3, on the other hand, shows higher significance than Ficolin 2. As shown in Figure 8A (KPC-Kp infection), ficolin 3 in AF treated with hA-MSCs is increased at 24 and 72 h and at 1 week compared to 0 h (24 h, *p* < 0.001; 72 h, *p* < 0.01; 1 week, *p* < 0.05). While untreated AF shows a lower ficolin 3 concentration only at 24 h (*p* < 0.05) and 1 week (*p* < 0.05). Figure 8B (E. coli-CR infection) shows, for both groups studied, a higher increase in ficolin 3 concentration compared to 0 h, at all times (AF group 24 h, *p* < 0.01; 72 h and 1 week, *p* < 0.001; AF plus hA-MSCs 24 h, *p* < 0.001; 72 h, *p* < 0.01; 1 week, *p* < 0.001).

## 3. Discussion

Cirrhosis involves changes in organ function with serious systemic consequences. The clinical manifestation is characterized by a first phase, called compensated cirrhosis, followed by a progressive phase of decompensation due to the increase in portal hypertension that causes ascites, bleeding from esophageal varices, and encephalopathy until liver failure. Disease progression can be accelerated by the development of other complications, such as renal dysfunction, hepato-pulmonary syndrome, refractory ascites, and spontaneous bacterial peritonitis (SBP) [35]. SBP is bacterial infection of AF, caused by the translocation of bacteria from the intestinal tract into the ascites, and is the consequence of impairment of the hepatic reticulo-endothelial system, immune system dysregulation, and the reduced opsonic activity of AF in decompensated liver cirrhosis [36]. Once diagnosis of SBP is confirmed, adequate empirical antibiotic treatment must be promptly initiated, otherwise a delay in treatment may result in a high chance of mortality [37]. In the past, an antibiotic prophylaxis, in particular with norfloxacin, was advised to prevent SBP [38]; however, it is currently not advised due to the emergence of multidrug-resistant microorganisms in up to 52% of cirrhotic patients affected by bacterial infections [39,40] and worse cases are linked to the emergence of XDROs, which present higher risks of developing acute and chronic liver failure and lower infection resolution rates [41,42]. Thus, it is crucial to develop alternatives to antibiotics to prevent such complications [43].

In recent years, the demand for regenerative medicine approaches, such as nanotechnology and stem cell therapy, has been driven by the increase in degenerative and chronic diseases [44]. Among the several clinical applications of stem cells, promising results have been obtained in animal studies in the treatment of infectious diseases by using mesenchymal stem cells (MSC) microvesicles [45]; in particular, by increasing macrophage phagocytosis in E. coli infections [46,47]. However, the therapeutic mechanism and efficacy of MSC or their products may be different and specific for each pathogenic condition [43,48,49].

Increasing evidence confirms the role of stem cells (or their products) in counteracting systemic viral and bacterial infections by sustaining and restoring a compromised immune system. In particular, mesenchymal stem cell therapy has been used in clinical trials during the ongoing SARS-COV2 pandemic, aiming to mitigate multi-organ failure, and interesting results have been found as the reduction of proinflammatory cytokines and improvements of chest scans and clinical data [50]. Recently we have shown a therapeutic role of hA-MSCs in reducing portal pressure, improving liver microcirculatory function, and liver function tests in cirrhotic rats [51]. We also demonstrated that hA-MSCs do not undergo changes in contact with non-infected AF, but exploit their anti-inflammatory and condition-restoring capacity [13]. The aim of this work was to further investigate if hA-MSCs have a role in treating spontaneous bacterial peritonitis due to XDROs [52]. We conducted preliminary experiments by contaminating the AF of cirrhotic patients undergoing paracentesis with two carbapenem-resistant Enterobacterales (E. coli-CR and KPC-Kp strains), both isolated from urinary tract infections from our inpatients. In particular, this type of analysis allowed us to better understand the effect of hA-MSCs on the phagocytic capacity of monocytes of the WBC component of ascitic fluid in culture, and to investigate which complement system proteins are involved in the first defense response, and the effects of hA-MSCs on bacterial proliferation over time.

We demonstrated that hA-MSCs added to AF significantly reduce the proliferation of both bacterial strains at 24 h. The same effect does not occur in Control 2. The subsequent decrease in the bacterial load persists until 72 h in the case of E. coli-CR infection, while there seems to be no clear distinction at the same times between samples of ascites treated and without MSCs, in presence of KPC-Kp. It could be probably explained by the different proliferation kinetics of the two bacterial strains with KPC-Kp that reach a peak at exactly 24 h, and, in the same frame time, the presence of hA-MSCs is more effective. Regarding the macrophage polarization status at different time points, we noted that in in case of E. coli-CR infection, the M1 and M2 populations increase significantly according to time up to 72 h, compared to 24 h, while in case of KPC-Kp infection, the increase in M1 and M2 is more consistent within 24 h and the M2 component increases more at 72 h in comparison to AF without hA-MSCs. These results support the hypothesis that mesenchymal stromal cells obtained from human placenta have a rapid and efficient anti-inflammatory/immunomodulatory action in reducing bacterial load.

In our previous study, we evaluated the pro- and anti-inflammatory molecules in terms of released cytokines and molecules capable of stimulating the cells of the immune system [13], and we found that hA-MSCs viability was not affected by AF and, interestingly, hA-MSCs diminished pro-inflammatory cytokine production and promoted anti-inflammatory M2 macrophage polarization. Moreover, we found that there was no simultaneous significant decrease in the M1-like component, allowing continual phagocytosis activity of macrophages and NK cells to restore physiological conditions.

In this study, we also focused on proteins of the complement with proven important roles in infective clinical situations [53], and, in particular, based on recent studies that show how variations in the lectin pathway could be implicated in the development of liver cirrhosis, although the mechanism that mediates such a process is not fully understood [10]. We then evaluated the levels of some complement proteins in infected ascites in the presence of hA-MSCs. The complement system is composed of over 50 proteins, present both in the blood and lymphs, and can be found circulating, membrane-bound, and intracellularly. One of the proteins that we analyzed was C3a, which plays an inflammatory role and we found that it significantly reduced in presence of KPC-Kp (but not in the case of E. coli-CR) and hA-MSCs compared to ascitic medium cultured without stem cells at 24 and 72 h and at 1 week.

Mannose-binding lectin (MBL) is an important mediator of innate immunity and is synthesized primarily by the liver. Recent studies highlighted the possibility that high levels of the MBL protein can make the environment more susceptible to infection and disease [54]. For this reason, in this work, we evaluated MBL levels when subjected to bacterial stimuli, compared to the amount of protein present in the ascitic fluid post paracentesis. In particular, the results obtained showed a lower concentration of MBL protein in AF treated with hA-MSCs compared to AF cultured without hA-MSCs at 1 week following KPC-Kp or E. coli-CR infection, compared to time T0.

Instead, regarding ficolin concentration, ficoline 2 did not show a particular involvement in both types of bacterial infections, while there was a significant increase in the production of ficolin 3 at 24 and 72 h following infection with KPC-Kp.

The limitations of our study were that the experiments were done in vitro, which give us a partial vision of the complex crosstalk between hA-MSC, cellular and humoral component of the infected AF.

In conclusion, the data obtained in this study show, for the first time, the potential of hA-MSCs in treating infection of ascites, obtained from decompensated cirrhotic patients, as shown by bacterial load, reduction, changes in M1-M2 polarization status, and complement proteins production. In particular, in presence of E. coli-CR and hA-MSCs the bacterial proliferation reduces at each time point, up to 1 week, when compared to the control without hA-MSCs; M1 and M2 components both increased at 72 h and in the meantime a MBL protein concentration decrease was noted. Instead, in the presence of KPC-Kp, a prominent and significant antibacterial effect was evident at 24 h and was then maintained up to 1 week, but without significance when compared to controls; a better response in terms of M1 and M2 component increase, reduction of inflammatory protein C3a concentration, and reduction of MBL at 1 week, and an increase of ficolin 3 at 24 and 72 h compared to AF samples without hA-MSCs, were shown.

These findings suggest that hA-MSCs can be considered a new strategy for a therapy against ascitic fluid infection and, in particular, in presence of carbapenem-resistant Enterobacterales. The next important step will be to explore, through a proteomics approach, which molecules present in the hA-MSCs secretome are responsible for the antibacterial and anti-inflammatory effects of hA-MSCs and then further validate the results through an animal model with SBP.

## 4. Materials and Methods

### 4.1. Patients and Ascitic Fluid Collection

AF was obtained from two cirrhotic patients (1 male and 1 female), mean age 69 (range 60–78), in Child B-C class (according to Child–Pugh score) complicated by refractory ascites. Liver cirrhosis etiology was related to non-alcoholic steatohepatitis (NASH). Patients were admitted at IRCCS ISMETT (Mediterranean Institute for Transplantation and Advanced Specialized Therapies), in Palermo, Italy, and underwent standard care to routine paracentesis. Informed consent was obtained from both patients to participate in the study. AF was collected during paracentesis and signs of infection were excluded (polymorphonuclear leukocyte count in the AF was <250 cells/mm^3^ and AF culture resulted negative after 5 days). An amount of AF ranging from 1 to 2 L was collected from each patient, as explained above.

### 4.2. Isolation and Culture of Human Amnion-Derived Mesenchymal Stromal Cells (hA-MSCs)

The hA-MSCs were isolated, within 6 h after birth, from the amnion of human term placenta from 3 healthy donors at 36–40 weeks of gestation. Informed consent from all donors was obtained according to the tenets of the Declaration of Helsinki and institutional ethical regulations (IRRB/29/18, ISMETT Institutional Research Review Board). Amniotic membranes were manually separated from the chorion and washed several times in 0.9% sodium chloride (NaCl) containing 1% penicillin/streptomycin (P/S, 100 U/mL/100 µg/mL) (Sigma-Aldrich, MI, Italy) and 2nML-glutamine (Sigma-Aldrich). They were cut into smaller pieces of 3 × 3 cm, which were decontaminated with a brief incubation in 0.9% sodium chloride (NaCl) containing 1% P/S and 2 nM L-glutamine and 2.5% Esojod (Esoform, Italy). The pieces were then washed for 3 min in a solution of phosphate-buffered saline (PBS) (Biowest), 1% P/S, 12.5 mg/mL amphotericin B, 1.87 mg/mL cefamezin (Pfizer, Italy) and 5 min in PBS 1% P/S. After that, decontaminated fragments were incubated for 9 min at 37 °C in Hank’s balanced salt solution (HBSS, Lonza, CH) containing 2.5 U/mL dispase (Corning, NY, USA) and subsequently in Roswell Park Memorial Institute (RPMI) 1640 medium (Sigma-Aldrich) supplemented with 10% heat-inactivated fetal bovine serum (FBS) (Sigma-Aldrich), 1% P/S, 2 nM L-glutamine (complete RPMI 1640) for 5 min at room temperature (RT). Then, the fragments were digested with 0.94 mg/mL collagenase A (Roche, Germany) and 20 mg/mL DNase (Roche, Germany) for 2.5 h at 37 °C, and later filtered with both 100- and 70-µm cell strainers (BD Falcon, NJ, USA).The sieved cells were pelleted by centrifugation at 150–300× *g* for 10 min and resuspended in complete RPMI 1640. After isolation, we evaluated the yield, viability, morphology and primary cell culture growth. In order to evaluate the purity of the final pellet, we analyzed some of the markers commonly used for the detection of MSCs. The hA-MSCs were cultured at approximately 1 × 10^5^ cells/cm^2^, using Chang Medium (Irvine Scientific) supplemented with 1% L-glutamine and 1% P/Sat 37 °C and 5% CO_2_. Cell growth was monitored under inverted phase contrast light microscope (Olympus BH-2, Olympus Optical Co., Tokyo, Japan). The attached cells reached confluence 10 days after plating (passage 0). The cells were then trypsinized with a 0.05% trypsin/0.5 mM EDTA solution (Euroclone) and expanded into a T-75 flask for two passages. For our experiments, we used cells at passage two.

### 4.3. Bacterial Strains

In this work, we used K. pneumoniae carbapenemase KPC-producing strain, mentioned above as KPC-Kp, and an E. coli NDM-1/OXA-48 strain, described in the text above as E. coli-CR. These strains were collected from in-patients at our hospital and were previously characterized, including their multidrug resistance phenotype, elsewhere [55,56].

Both bacterial strains were grown overnight in MacConkey broth (Sigma) at 37 °C with agitation. In the following day, bacteria was enumerated using a UF-5000/4000 flow cytometer (Sysmex, Landskrona, Sweden), and dispensed in 24-well plates containing both hA-MSCs only in complete RPMI at a proportion 1:5 and hA-MSCs in co-culture with white blood cells (WBCs) from AF, at a proportion of hA-MSCs/WBCs of 1:5-WBCs/bacteria 1:1. The bacteria/cell co-cultures were incubated at 37 °C in a 5% CO_2_ atmosphere, and the optical density (OD) of the media was read at 600 nm at 1 h, 24 h, 72 h, and 1 week after plating. The time point of 1 h was considered our starting point for data analyses.

### 4.4. Exposure of hA-MSCs to Infected Ascitic Fluid

hA-MSCs were cultured for 1 h, 24 h, 72 h, and 1 week with AF infected with two carbapenem-resistant Enterobacterales (as described above) in order to evaluate their antibacterial capacity. As control, we used hA-MSCs grown in complete RPMI 1640 without penicillin/streptomycin infected with E.coli-CR and KPC-Kp. The cells were subjected to different assays in order to evaluate the final bacterial load and analysis of macrophage polarization. All conditioned media were decanted from the wells to a tube, collected, and stored at −80 °C until use for subsequent proteomic analyses. All experiments were done in triplicate, using two different AF samples.

#### 4.4.1. Analyses of Macrophage Polarization WBCs from Infected Ascetic Fluid in Co-Culture with hA-MSCs

The hA-MSCs were co-cultured with white blood cells (WBCs) obtained from the AF from 2 cirrhotic patients. The effects of hA-MSCs on the phagocytic capacity of the cellular components present in the AF after bacterial infection was evaluated. The WBCs were obtained after centrifugation of the initial ascites at 300× *g* for 10 min. The red blood cells were lysed through 10 min of incubation with 1X erythrocyte lysis solution (10X Stock Solution: 41.4 g NH_4_Cl, 5 g KHCO_3_, 1 mL EDTA 0.5 M pH 8, in 500 mL double distilled H_2_O) and subsequent centrifugation at 300× *g* for 10 min. Total WBCs were counted with an XN-3000 cell counter (Sysmex Landskrona, Sweden) and the cell pellet was resuspended in an appropriate volume of ascites for each respective patient. The hA-MSCs (7.5 × 10^4^) were plated in 24-well plates (Corning) using RPMI and incubated at 37 °C and 5% CO_2_ for 12 h, after which 3.8 × 10^5^ WBCs were plated (hA-MSCs/WBCs ratio 1:5) and co-cultured for 1 h, 24 h, 72 h, and 1 week. In order to evaluate macrophage stimulation, the experiments were carried out with the addition of E.coli-CR and KPC-Kp (WBCs/bacteria ratio 1:1) in the presence or not of hA-MSCs. WBCs only resuspended in AF and WBCs resuspended in AF in co-culture with hA-MSCs only (without bacterial infection) were used as controls. hA-MSCs grown in RPMI (private of P/S) at the same time points, with and without bacterial infection, were used as controls. After culturing, WBCs were separated from ascites and harvested, washed twice with FACS buffer, and analyzed by flow cytometry, as described above, to evaluate the state of macrophage polarization. The cells were stained for the detection of M1 (CD14+ CD16+) and M2 (CD14+ CD206+) (BD Biosciences, Brea, CA, USA) profiles (Table 1). In the same experimental conditions was evaluated the variation of surface marker expression on hA- MSCs alone stimulated with bacteria for the same time points (data not shown).

#### 4.4.2. Evaluation of Bacterial Load in Ascitic Fluid

A UF-5000 flow cytometer (Sysmex, Landskrona, Sweden) was used to evaluate the hA-MSC treatment effects on the bacterial load at different chosen times. The hA-MSCs (7.5 × 10^4^) were plated in 24-well plates (Corning) using RPMI and incubated at 37 °C and 5% CO_2_ for 12 h, after which 3.8 × 10^5^ WBCs were plated and co-cultured for 1 h, 24 h, 72 h, and 1 week with the addition of E.coli-CR and KPC-Kp, as previously described (all the experiments were carried out in triplicate).

#### 4.4.3. Phagocytosis Assay

Phagocytosis activity was measured in both WBCs alone and WBCs co-cultured with hA-MSCs by administering E. coli conjugated to a pH-sensitive fluorescent dye (Bioparticles^®^). Bioparticles^®^’s fluorescence was increased at low pH when in phagocytic lysosomes. The phagocytic function was investigated by incubating the cells with fluorescent-labeled Escherichia coli bioparticles and analyzed by flow cytometry and fluorescence microscopy. The amount of Bioparticles^®^ phagocytosed was determined by measuring fluorescence intensity using an EVOS M5000 cell imaging system (Thermo Fisher, Waltham, MA, USA). Phagocytosis of the particles by WBCs was measured by flow cytometry using a BD FACS CelestaTM SORP instrument (15 colors). Analyses were carried out using FACS CelestaTM SORP flow cytometer and FACS Diva software version 9.0 (BD Biosciences, Brea, CA, USA). The hA-MSCs were plated in a black 96-well Costar Assay Plate with a clear flat bottom (Corning) at a seeding density of 45,000 cells/well using RPMI and incubated overnight at 37 °C and 5% CO_2_. The starting time point was established when the cells were exposed to 100 μL of AF containing 10,000 WBCs/well for imaging assay and 100,000 WBCs/well for flow cytometry assay. Each culture condition was carried out and incubated for 1 h, 24 h, 72 h, and 1 week at 37 °C and 5% CO_2_. At the different time points chosen, according to the instructions, 100 μg/mL of fluorescent particles were added and the generated fluorescent signal was found to be proportional to the amount of phagocytosis.

#### 4.4.4. Assessment of the Rate of C3a and Mannose Binding Lectin

Evaluation of complement protein activation after contact with bacteria was studied by measuring the presence of MBL and C3a in the condition media over time, compared to the amount of protein in the AF after paracentesis.

The levels of the complement proteins involved in two different stages of defense against bacteria were determined in each conditioned AF for all different time points, after culture with hA-MSCs or without hA-MSCs (as control) using magnetic bead technology from Luminex with the C3a Human ProcartaPlex™ Simplex Kit (Thermo Fisher, Waltham, MA, USA) according to the manufacturer’s instructions. The data were acquired using xPONENT^®^ 3.1 software for Luminex 100/200 (Luminex Corporation, Austin, TX, USA). Concentrations of each protein were calculated by interpolation from standard curves. Results are shown as a fold decrease of each conditioned medium relative to basal protein compositions of the two post-paracentesis (*n* = 6). ProcartaPlex™ Analyst 1.0 was used to analyze the obtained data.

#### 4.4.5. Ficolin-3 and Ficolin-2 Enzyme-Linked Immunosorbent Assay

Ficolins are innate immunity pattern recognition molecules that bind to distinct pathogen-associated molecular patterns (PAMP) by facilitating phagocytosis and activation of complements through the lectin route [57,58].

For the detection of ficolins by enzyme-linked immunosorbent assay (ELISA), we used a recombinant human Ficolin-2 and Ficolin-3 (Invitrogen) as capture and a Human Ficolin-2 and Ficolin-3 Biotin Conjugated detection antibody (Invitrogen). As secondary reagents, we used Streptavidin-HRP and TMB Substrate (Invitrogen, EH192RB, EH193RB), according to the manufacturer’s instructions. The standard curve concentration points, as well as the samples were tested in duplicate on a single plate. Two different concentration values were obtained for each sample. The acquired data were analyzed using a Spark^®^ Multimode microplate reader (Tecan Trading AG, CH). The plates were measured at 450 nm. All samples and standards were analyzed in duplicate (*n* = 6).

## 5. Statistics

All experiments were done as three independent experiments using two different AF samples from two different donors. Data from different groups were compared using an unpaired *t*-test, while data from the same groups were compared using the 1-h or 24-h time point as “time zero”, and applying a paired *t*-test. Results are expressed as the mean ± standard deviation (SD). Differences were considered statistically significant at *p* < 0.05.

## Figures and Tables

**Table 1:** List of antibodies used for flow cytometry analysis.

*Antigen*	*Clone*	*Isotype*	*Dilution*	*Conjugation*	*Manufacturer*
*CD14*	*MφP9*	*Mouse BALB/c IgG2b, κ*	*1:20*	*APC Cy7*	*BD Biosciences, CA, USA*
*CD16*	*B73.1*	*Mouse BALB/c IgG1, κ*	*1:20*	*PE*	*BD Biosciences, CA, USA*
*CD206*	*19.2*	*Mouse IgG1, κ*	*1:20*	*APC*	*BD Biosciences, CA, USA*

## Data Availability

The data that support the findings of this study are available from the corresponding author, M.P., upon reasonable request.
